# Effectiveness of Solution-Focused Brief Therapy (SFBT) on Depression and Perceived Stress in Patients with Breast Cancer

**Published:** 2018-10

**Authors:** Angham Aminnasab, Samaneh Mohammadi, Maryam Zareinezhad, Tania Chatrrouz, Seyedeh Bita Mirghafari, Soheila Rahmani

**Affiliations:** 1 Department of Psychology, Islamic Azad University, Kish International Branch, Kish Island, Iran,; 2 Department of Clinical Psychology, Faculty of Psychology and Educational Sciences, Shiraz University, Shiraz, Iran,; 3 Department of Clinical Psychology, Islamic Azad University of Tehran, North Branch, Tehran, Iran,; 4 Department of Clinical Psychology, Islamic Azad University, Sari Branch, Iran,; 5 Department of Clinical Psychology, Islamic Azad University, Tonekabon Branch, Iran; 6 Department of Psychology of Health, Islamic Azad University, Karaj Branch, Karaj, Iran.

**Keywords:** Breast cancer, Depression, Perceived stress, Solution-focused brief therapy (SFBT)

## Abstract

**Background:**

Attending to psychological status in patients with breast cancer, because of expanded damage and mortality in these patients, is important. The present study investigated the effectiveness of Solution-Focused Brief Therapy (SFBT) on depression and Perceived Stress in Patients with breast cancer.

**Materials and Methods:**

This research was a semi-experimental with pretest, post-test and follow-up (1 month), which was conducted from November to February, 2016. In this study, 30 patients with breast cancer who attended Imam Hossein Hospital in Tehran city were selected by convenience sampling method and randomly were assigned in 2 experimental (n=15) and control groups (n=15) and Cohen’s Perceived Stress Scale and Center Epidemiological Studies Depression Scale were administrated as pretest. Experimental group received 8 sessions of Solution-Focused Brief Therapy SFBT and control group received no intervention. At the end, post-test was administrated on two groups and, repeated measure multi-variable method was used for data analysis by SPSS-21 software.

**Results:**

The results of the present study indicated that there were significant differences between the experimental and control groups after administrating SFBT. Thus, the mean of depression and perceived stress of experimental group decreased (P<0.001).

**Conclusion:**

The result of study that showed SFBT is effective in decreasing depression and perceived stress in patients with breast cancer. Therefore, in order to improve the positive psychological state in these patients psychological screening must be performed and if needed clinical trials and appropriate intervention be considered.

## INTRODUCTION

The increased prevalence of cancer in recent years and its impact on various aspects of physical, psychological and social life have led to cancer being recognized as a major health problem of this century (1). Cancer is one of the most common non-infectious diseases caused by an impairment in the rate of cell proliferation and differentiation, and by attacking healthy tissues of the body causes severe illness and, as a result, death (2). Types of cancers are a wide range of diseases, each of which has its own etiology, therapeutic, and prognostic basis (3).

The most common and most influential type of cancer among women is breast cancer, emotionally and psychologically (1), with a prevalence of five year-survival rate of about 80 to 95% in women (4). Breast cancer is not considered as a deadly disease today and is increasingly recognized as a curable disease (5). However, the process of diagnosis and treatment of this disease is highly stressful (4). Long-term stress causes an increased risk of depression (6). Psychological factors such as depression increase the mortality rate of patients with breast cancer. The prevalence of depression in women with breast cancer is estimated to be more than 37% (4). Many women with breast cancer report depression symptoms that are very serious, but are below than threshold rate of symptoms for major depression diagnosis (6). However, in either case, depression symptoms are highly effective in breast cancer symptoms. The main symptom of depression in women with breast cancer is an overwhelming fear of recurrence and metastasis (7). Indeed, the suffering from the disease, the concern of the future of family members, the fear of death, the complications of treating the disease, reducing the amount of daily functions, mental impairment and sexual problems are some of the important factors that predispose the continuation of depression in these patients (1, 8).

Also, another psychological variable that appears to have a significant role in the prediction of suicide in the woman with breast cancer is perceived stress (9). Stress is a condition in which biological, psychological, and environmental factors interact (10). Other study has shown that stress can lead to rapid progression of cancer disease and severe physical and psychological consequences for patients (11). Perceived stress is a mode that reflects the overall assessment of the importance and severity of environmental and personal challenges. Therefore, these individual and environmental factors are important in understanding the stressors (10). Considering the many negative effects that depression and stress have in the process of the disease and the trend of breast cancer, conducting researches is needed to identify the effective factors in reducing these psychological variables.

One of the most effective therapeutic interventions to help people who suffer from stress and depression is Solution-Focused Brief Therapy (SFBT) (12). This therapy was initiated in the early 1980s at Brief Family Therapy Center in Milwaukee. SFBT was developed by two social workers called Steve De Shazer and Insoo Kim Berg and their colleagues who sought to investigate short-term therapeutic effects and techniques for helping patients change (13).

Although the history of SFBT is short, in recent years, this approach has become popular among counselors and mental health professionals around the world (14). The SFBT has gained great popularity due to the emphasis on rapid changes in treatment and respect for the views of both those who are consistent with the philosophy of health care and, like all medical interventions, help the authorities to create solutions which produce a more positive quality of life. Research has shown that even in emergency situations it can produce good results.

Given what has been said, it seems that SFBT is important for improving the depression and perceived stress of patients with breast cancer. Therefore, the present study was conducted with the aim to evaluate the effectiveness of SFBT on depression and perceived stress in patients with breast cancer.

## MATERIALS AND METHODS

The present study was a semi-experimental with pre-test, post-test, and control group. The statistical population of this study was all patients with breast cancer referring to Imam Hussein Hospital in Tehran during the study. In order to calculate the sample size, according to the fact that the proper sample size was 15 people for each group (15), the sample size was selected equal to 15 people for each group.

The inclusion criteria to this study included diagnosis of breast cancer stages 1, 2, and 3, based on clinical findings and cytology studies and diagnosis of a physician, informed consent and willingness to participate in the research, the ability to attend meetings and collaborate on tasks, willingness to cooperate in completing scales, physical and psychological stability (lack of significant physical or psychological symptoms that intervene during sessions, such as recurrence of disease or the development of metastasis at different points in the body during the study), depression score higher than average, duration of breast cancer diagnosis more than a month, the level of education higher than fifth grade, and age range between 30 and 55 years. Exclusion criteria from the present study included other types of cancers, under treatment due to another physical or psychological illness, cognitive impairment or weakness in cognitive function, the presence of severe symptoms of the disease in such a way that make the participation of patient to study difficult or almost impossible and presence of other psychological symptoms (such as anxiety).

In this way, among patients with breast cancer who had treatment documents in Imam Hussein Hospital in Tehran, some were randomly selected and assigned into two groups of experimental and control groups if they had the inclusion criteria.

The research method was in this way that to investigate the effectiveness of SFBT on depression and perceived stress in patients with breast cancer, a pre-test, post-test design and follow-up was used that included the following executive procedures.

At first, among the patients referring to Imam Khomeini Hospital, who had a treatment document and referred to a specialist for regular visits on specific dates, 30 people with inclusionary criteria were selected. Then 30 sheets, that the letter A had been written on 12 sheets and B on the other 12 sheets, were prepared. During the pre-test, using a structured clinical interview (SCID), the Cohen’s Perceived Stress Scale (PSS) and Center Epidemiological Studies Depression Scale for each patient individually, each patient was asked to select one sheet from the prepared sheets, and in this way all patients were randomly assigned to two groups of A and B. Then, randomly, one group was assigned to the control group and the other group was considered as SFBT group. Patients in the SFBT group received 1 session of 2 hours psychotherapy every week in the Oncology Department of Imam Khomeini Hospital in Tehran by a therapist (doctor of health psychology) who had completed the SFBT courses and was specialized in this field.

The protocol of sessions for SFBT is presented in table 1. The instruments used in this study were Demographic sheet sample, Cohen’s Perceived Stress Scale (PSS), and Center Epidemiological Studies Depression Scale.

**Table 1.** Structure of intervention sessions of solution-focused group therapy

**Session**	**Topic**
**First session**	Explain the principles of the therapeutic sessions, Introduction and expression of the aims of sessions, introducing the problem and method of writing reports tor tasks. Setting the goals in a positive, specific, tangible, and measurable way.
**Second session**	Understanding the approaches for coping with problems from different Psychological perspectives
**Third session**	Omission of the disruptive behavior and cognitive patterns by the use of the miracle question, using the targeting technique
**Fourth session**	Determining situations and solutions, using the technique of exploring exceptions in reducing problems and detecting the moments when the problems and complaints are less for understanding the existence of positive exceptions in life, increasing hope, and reducing the level of problems.
**Fifth session**	Realizing own abilities when needed. Using the master key technique: doing a different task (preparing a list of problems and providing different solutions)
**Sixth session**	Learning new ways of thinking, feeling, acting and behaving, and experiencing new feelings by the use of the very significant word “instead.” Using the master key technique: paying more attention to the pathological cognitive and behavior and the results of behavior and cognitive
**Seventh session**	Clear understanding of the participants, of changes made in their lives by themselves, and realization of the personal skills they have used in the process. Familiarizing with master key technique: writing thoughts, reading them and then burning them, writing negative messages and replacing them with positive messages.
**Eighth session**	Providing a summary about the subject matters of held sessions and a review about them, answering to all of the questions and uncertainties of group members, implementing the posttest.

### Demographic sheet sample

This sheet sample included the age, sex, level of education and marital status of the patient, which was provided and assessed by the researchers in this study.

### Cohen’s Perceived Stress Scale (PSS)

This tool was developed by Sheldon Cohen in 1983 and has 3 versions with 4, 10 and 14 items used to measure perceived general stress over a past month. Each article is graded based on the 5-degree Likert range from never (0 score), to very often (4 scores), and questions 4, 5, 7, 9, 10 and 13 are reversely scored. Finally, the score will be between 0 and 56. A higher score represents more perceived stress (16). Cronbach’s alpha has been considered equal to 0.84, 0.85 and 0.86(17).

### Center Epidemiological Studies Depression Scale (CES-D)

The Center for Epidemiology Studies used the Depression Scale (CES-D) to assess depressed mood, which is a self-report questionnaire with 20 items which is designed for assess symptomatic depression scale. Respondents graded their depression on a 4-point scale to the extent that they experienced signs last week. The probable range score of this questionnaire is from 0 to 60, and higher scores are more signs of depression. Internal consistency reliability (Cronbach’s alpha) of this questionnaire had a range from 84 to 90% in previous researches. Psychometric investigations for CES-D yielded convergent and differential validity. Also, for this scale, proper credibility and structure factor have been found among different cultural groups (18). This scale has been used in various researches in Iran and its reliability and validity have been reported to be good. For example, in the research by Bakhtiarpoor et al. (19), reliability coefficients of depression questionnaire CES-D have been reported using Cronbach’s alpha to be equal to 91%. In terms of validity, the correlation of this test with Beck Depression Inventory was significant (r = 90% and P =0.0001).

To analyze the research data, descriptive statistics such as mean, standard deviation and frequency and inferential statistics such as Multivariate Repeated Measurement Variance Analysis (ANOVA) were used using SPSS software version 21.

## RESULTS

The demographic characteristics of the samples in this study are presented in table 2.

**Table 2.** Demographic characteristics of the studied subjects

		**Experimental**		**Control**	

		Frequency	Frequency percentage	Frequency	Frequency percentage
**Age**	20 to 25	2	13.3	3	20
	26 to 30	4	26.7	2	13.3
	31 to35	2	13.3	3	20
	35 to 40	5	33.3	2	13.3
	41 to 45	2	13.3	5	33.3
**Mean and standard deviation**		7/69±32/67		60/03±34/80	
**Education**	High school	7	46.7	7	46.7
	Diploma	6	40	7	46.7
	Higher than diploma	2	13.3	1	6.7
**Marital status**	Married	10	66.7	11	73.3
	Single	5	33.3	4	26.7

The mean and standard deviation of depression and perceived stress variables in three pre-test, post-test and follow-up situations according to the experimental and control groups are reported in table 3.

**Table 3.** Mean and standard deviation of variables of depression and perceived stress in patients with breast cancer in terms of time (pre-test, post-test, follow-up)

	**Experimental group (n =15)**			**Control group (n =15)**		
**Variables and test dimensions**	**Pre-test**	**Post-test**	**Follow-up**	**Pre-test**	**Post-test**	**Follow-up**
**Depression**	43.07±5.09	29.60 ± 5.11	31.60±4.38	42.93±4.81	41.67±4.38	43.07±4.49
**Perceived stress**	35.27±3.78	23.53±4.91	24.73±5.28	34.40±4.59	33.60±4.10	34.73±4.31

As shown in table 3, not only the mean score and standard deviation of depression have been decreased from the pre-test to post-test, but have been relatively stable at the follow-up stage. Also, the mean and standard deviation of perceived stress has decreased from the pre-test to post-test and has a relative stability in the follow-up stage.

In this study, statistical analysis of depression and perceived stress scales was performed in two experimental and control groups with Multivariate Repeated Measurement Covariance Analysis. In this analysis, the pre-test scores were considered as auxiliary variables, and post-test scores and follow-up scores were considered as dependent variables and time was considered as a moderator variable. Prior to the analysis, the preconditions for using the model were first examined.

As can be seen in table 4, the zero assumption for the equality of the variances of the two groups is confirmed in the depression and perceived stress variables. That is, the variances of the two groups in the society are equal and do not have a significant difference. So, given Levine’s observation, it is possible to run a variance analysis with repeated measurements of the results to study the research hypotheses.

**Table 4.** Results of Levine test on the assumption equation of sample groups’ variances

**Index**	**Levels**	**F**	**First degree of freedom**	**Second degree of freedom**	**Significance level**
**Depression**	Post-test	0.322	1	28	0.575
	Follow up	0.212	1	28	0.278
**Perceived stress**	Post-test	0.766	1	28	0.389
	Follow up	1.057	1	28	0.313

The results of Mauchly’s test for the depression variable show that there is no equality assumption for the covariance matrix (P = 0.003, df = 2, Mauchly’s W = 0.651). Therefore, due to the inequality of the variance-covariance matrix, the Huynh-Feldt test was used.

The values of this test showed that the test related to the main effect of time (P> 0.001, Partial Eta Squared = 0.880, F=206.156) and the interaction of time and groups (P> 0.001, Partial Eta Squared = 0.854, F = 163.828) between the groups are significant. The results of multivariate tests showed that the effect of time factor (pre-test, post-test, follow-up) was significant for depression variable (Partial Eta Square=0.912, P<0.001, F=140.353, Pillai’s Trace =0.912). Also, the interaction of time × group was significant (Partial Eta Square=0.881, P<0.001, F=99.559, Pillai’s Trace=0.881). Considering the fact that the difference between the two experimental and control groups was significant and according to the results of table 3, the mean of the experimental group in the post-test and follow-up stage was lower than the control group, it can be stated that the independent variable (SFBT) has been effective in reducing depression.

**Figure:**
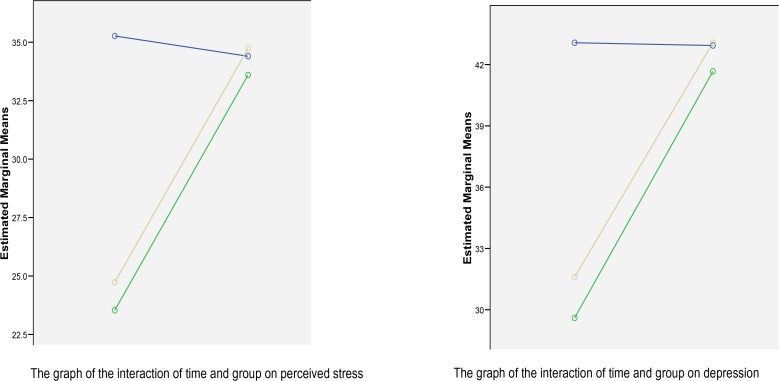


## DISCUSSION

According to the aim of this study regarding the effectiveness of SFBT on depression and perceived stress in patients with breast cancer, the results of repeated multivariate analysis of variance showed that SFBT leads to a reduction in depression and perceived stress in the patients with breast cancer. This finding that SFBT leads to a reduction in depression in patients with breast cancer is consistent by the results of the study by Dashtizadeh et al. (13), Abbasi et al. (20) and Wichowicz et al. (21) in different samples and researches.

Dashtizadeh et al. (13), in explaining their study, state that SFBT differs from many other therapies in that the therapist is not claimed to be a specialist. This is a different way of thinking about the help process. Therapists of SFBT assume that change is sustainable, and clients have their own initiative to overcome problems, and also focus on discovering solutions rather than problem solving. According to the principle (problem/exception) in SFBT, depression is conceptualized as (depression/non-depression). In other words, depression necessarily has a distinct point, that is, times when the person is not depressed. In solution-based therapy, it is assumed that depression largely preserves itself, as the clients see it as a permanent and continual occurrence. The theory of change in the solution-based therapy believes that if the client can identify and reinforce exceptions for the problem, it can result in profound change (that is, exceptions become laws). In the solution-based therapy, there is the idea that clients with good coping skills, abilities, and pre-existing resources and experience (exceptions) can better reduce their symptoms of depression and other clinical symptoms. In solution-focused therapy, it is assumed that if these positive points (exceptions) are identified and strengthened, a significant change will occur in the reaction of the clients towards depression.

Also, the finding that SFBT reduces the perceived stress of patients with breast cancer is consistent with the results of Wichowicz et al. (21), Shakarami et al. (22) and Beauchemin (23) in the different samples and researches.

In explaining this finding, it can be argued that therapists of SFBT use many techniques that reduce stress. One of these techniques is the use of grading techniques. Most grading questions are used to measure progress during therapeutic sessions. Grading questions want clients to rank their status or goal with scales from 1 to 10. For example, at the beginning of the therapy, clients are asked to determine the severity of their stress on the degree scale. After several sessions of therapy, the clients indicate their problem status on a grading scale, and in this way, the clients review their severity of problems during each degree of gradation. This technique helps the authorities to understand the extent of progress and improve their condition in the treatment sessions.

This study has some limitations and attention to them is important in generalizing the results. Among them, lack of sufficient samples that meet the inclusion criteria into the study for the third and fourth groups and comparison of other therapies, is therefore recommended in future research to focus on comparisons of new psychological treatments to reduce the psychological symptoms of patients with cancer.

Also, other groups must be assessed with drug therapy and placebo in addition to the experimental and control groups in order to provide more comparisons. Among other limitations of the present study, is the lack of long-term follow-up (1 year or more) due to the lack of access to all participants over time. Therefore, researchers must design studies with the aim of examining long-term effects of such interventions.
